# Recent Advances in Metabolomics and Lipidomics Studies in Human and Animal Models of Multiple Sclerosis

**DOI:** 10.3390/metabo14100545

**Published:** 2024-10-13

**Authors:** Petros Pousinis, Olga Begou, Marina Kleopatra Boziki, Nikolaos Grigoriadis, Georgios Theodoridis, Helen Gika

**Affiliations:** 1 Department of Chemistry, Aristotle University of Thessaloniki, 54124 Thessaloniki, Greece; ppousin@chem.auth.gr (P.P.); mpegolga@chem.auth.gr (O.B.); gtheodor@chem.auth.gr (G.T.); 2 Biomic_AUTh, Center for Interdisciplinary Research and Innovation (CIRI-AUTH), 57001 Thessaloniki, Greece; 3 Laboratory of Experimental Neurology and Neuroimmunology and the Multiple Sclerosis Center, 2nd Department of Neurology, AHEPA University Hospital, Aristotle University of Thessaloniki, 54636 Thessaloniki, Greece; bozikim@auth.gr (M.K.B.); ngrigoriadis@auth.gr (N.G.); 4 Laboratory of Forensic Medicine & Toxicology, School of Medicine, Aristotle University of Thessaloniki, 54124 Thessaloniki, Greece

**Keywords:** meta-analysis, multiple sclerosis, metabolomics, lipidomics, LC-MS, GC-MS, NMR, pathway analysis, biomarkers, review

## Abstract

Multiple sclerosis (MS) is a neurodegenerative and inflammatory disease of the central nervous system (CNS) that leads to a loss of myelin. There are three main types of MS: relapsing-remitting MS (RRMS) and primary and secondary progressive disease (PPMS, SPMS). The differentiation in the pathogenesis of these two latter courses is still unclear. The underlying mechanisms of MS are yet to be elucidated, and the treatment relies on immune-modifying agents. Recently, lipidomics and metabolomics studies using human biofluids, mainly plasma and cerebrospinal fluid (CSF), have suggested an important role of lipids and metabolites in the pathophysiology of MS. In this review, the results from studies on metabolomics and lipidomics analyses performed on biological samples of MS patients and MS-like animal models are presented and analyzed. Based on the collected findings, the biochemical pathways in human and animal cohorts involved were investigated and biological mechanisms and the potential role they have in MS are discussed. Limitations and challenges of metabolomics and lipidomics approaches are presented while concluding that metabolomics and lipidomics may provide a more holistic approach and provide biomarkers for early diagnosis of MS disease.

## 1. Introduction

Multiple sclerosis (MS) is a multifactorial chronic demyelinating disease of the central nervous system that has both inflammatory and neurodegenerative origins. It affects more than 2.5 million people worldwide [1]. Relapsing-remitting MS (RRMS) is the most common disease course, diagnosed in about 85% of the patients. Before approved disease-modifying therapies (DMTs) were available, studies indicated that 50% of those diagnosed with RRMS would transition to SPMS within 10 years, and 90% would transition within 25 years. While MS experts agree that disease-modifying treatments slow disease progression, it is too soon to tell the extent to which they alter or delay the transition to SPMS (https://www.nationalmssociety.org, accessed on 26 August 2024). Approximately 15% of MS patients develop a progressive course from the onset without relapses and remission stages, known as primary progressive MS (PPMS). Whether PPMS and SPMS are distinct phenotypes or they are variations of the same disease is a focus of extensive research [2]. Although the role of neuroinflammation in MS is well studied, the progressive neurodegenerative component of the disease (PPMS and SPMS) is yet to be fully characterized.

It has been shown recently that lipid metabolism is implicated in MS pathophysiology [3,4,5,6]. Lipids involved in myelin formation are of utmost importance in MS pathogenesis because of the demyelinating nature of the disease in the CNS [7]. In addition, although the exact etiology of the disease remains obscure, it is clear that alterations in the metabolome contribute to this process. The commonly perturbed pathways in MS and preclinical models of MS include nucleotide metabolism, amino acid metabolism, the tricarboxylic acid cycle, and the D-ornithine and D-arginine pathways, with a significant role in signaling and energy supply, reviewed here [8].

Over the last few years, many metabolomics [9,10,11,12,13,14,15,16] and lipidomics [17,18,19,20,21,22,23,24,25,26] studies in MS have been reported by our research group and others, on various human biofluids, such as plasma, serum, cerebrospinal fluid (CSF), tears, and urine, reviewed here [27,28,29].

Trepanier et al. [30] studied the cuprizone mouse model of demyelination and measured oleic acids and phosphatidylcholines (PCs) in the brain. It was found that oleic acid was decreased after cuprizone treatment and increased during the recovery stage when compared to control. Wheeler et al. [31] showed that levels of sphingolipids were attenuated and phosphatidylethanolamine (PE) were increased in human brain tissue from MS and control post-mortem cases using mass spectrometry shotgun lipidomics. Therefore, the previous literature suggests that alterations in the lipid profile in biological samples can drive the development of MS and CNS demyelination in humans and in respective animal models.

In this review, the latest metabolomics and lipidomics applications in human and animal models of MS-like disease and/or CNS demyelination in various biofluids are presented. Then, altered pathways among different sample types in human MS and animal cohorts are presented and discussed. At the end, limitations and challenges are presented to further explore future metabolomics and lipidomics approaches to better understand MS pathogenesis.

## 2. Results

### 2.1. Metabolomics and Lipidomics Studies in MS

In order to explore the latest metabolomics and lipidomics studies of MS, an advanced literature search was performed using the following words: **“Multiple Sclerosis [Title] AND metabolomics OR lipidomics [Title/Abstract]”, from January 2013 until January 2024.** This search returned 87 results. Experimental articles comparing findings from metabolomics and lipidomics analysis in biological samples of human MS cohorts and animal MS-like models are included here. Reviews, editorials, conference summaries, and communications articles were excluded.

### 2.2. Metabolomics- and Lipidomics-Based Analyses in MS Human Studies

Thirty-one articles were found that reported findings from metabolomics and lipidomics analyses on brain tissue and CSF [9,13,20,21,22,23,24,32,33,34] (Table 1), as well as in plasma or serum, urine, and tear samples from human cohorts [10,11,12,14,15,17,19,25,26,35,36,37,38,39,40,41,42,43,44,45] (Table 2). Amongst them, 9 articles used CSF samples alone, 1 article used brain tissue (normal-appearing white matter, NAWM), 1 article used CSF and plasma, 10 articles used only plasma samples, 8 articles used only serum samples, 1 article used tears and serum, and 1 article used only urine samples. The aim of these studies was to find biomarkers using metabolomics and lipidomics methods in the abovementioned biofluids between controls, clinically isolated syndrome (CIS), other neurological disease (OND), RRMS, PPMS, and SPMS patients. Human metabolomics and lipidomics studies are presented in next paragraphs and are summarized in Table 1 and Table 2.

#### 2.2.1. Brain Tissue and CSF

Exploration of MS necessitates a comprehensive understanding of the molecular dynamics within the central nervous system. Both CSF and brain tissue samples emerge as a pivotal avenue in unraveling the mechanism of the disease, as they are a rich source of molecular information, providing unique insights into the specific alterations and biomarkers associated with MS.Herman et al. [46], applied liquid chromatography high-resolution mass spectrometry (LC-HRMS) to investigate potential disease progression differentiation in the CSF metabolic profile of SPMS and RRMS patients and controls with other non-inflammatory neurological diseases. Citrulline, 5-Hydroxytryptophan, N-Acetylserotonin, kynurenate, 3-Methoxytyramine, and caffeine were significantly altered between SPMS and controls, while pipecolate, 3-Methoxytyrosine, biliverdin, 3-methoxytyramine, and caffeine were amongst the metabolites that were increased in SPMS compared to RRMS patients. In another study, the same group [13], by combing data obtained from magnetic resonance imaging (MRI) and protein and metabolite measurements of CSF, found 11 altered biomarkers that were able to significantly distinguish SPMS from RRMS patients. The levels of two metabolites, 20β-dihydrocortisol (20β-DHF) and indolepyruvate, were found statistically increased in the SPMS versus RRMS cohort. Kim et al. [32] also used the 1H-NMR analytical platform to explore possible CSF metabolic profile alterations amongst control, MS, and NMOSD cohorts. 2-hydroxybutyrate, acetone, formate, and pyroglutamate were found increased both at MS and NMOSD groups compared to controls, while acetate and glucose were decreased in the same comparisons. Citrate and lactate were found altered only in MS group or NMOSD group, respectively, when compared to healthy participants. In 2016, the group of Aeinehband [9] redirected their focus to one metabolic pathway and reported the first analysis of CSF kynurenine metabolites in MS patients of different disease states and in relation to neurocognitive symptoms. The study included two different cohorts of MS patients and controls. Levels of tryptophan, kynurenine, kynurenic acid, and quinolinic acid were determined in CSF samples by LC-MS/MS. When MS patients were stratified into RRMS relapse and RRMS remission patients, distinct patterns emerged. They revealed that absolute quinolinic acid levels and quinolinic acid/kynurenine and quinolinic acid/kynurenic acid ratios were increased in RRMS relapse patients compared to OND ones. Also, increased levels of tryptophan, kynurenic acid, and quinolinic acid were observed when the PPMS group was compared to OND. Interestingly, SPMS displayed the opposite trend for lower tryptophan and kynurenic acid compared to the OND group.

In addition to the comprehensive metabolome analysis, there is a focus on also scrutinizing the lipid content of CSF and brain tissue [20,21,22,33,34], sometimes complementary to the metabolic profile [23,24]. The lipid and metabolic profile of the CSF samples from MS patients and healthy controls was investigated using a complementary analytical platform of 1H-NMR spectroscopy [24]. MS patients exhibited lower levels of acetone, choline, urea, 1,3-dimethylurate, creatinine, and myo-inositol in comparison to controls. When analyzing the lipid levels in the CSF, saturated and unsaturated fatty acids (FAs), decreased in MS patients compared to controls. In the same manner, Pieragostino et al. [23] combined untargeted lipidomics MALDI-TOF-MS and targeted metabolomics LC-MS/MS for the analysis of the lipid profile, amino acids, and acylcarnitines in the CSF samples of MS and OND patients. In total, 10 metabolites were significantly altered between the two groups, including the up-regulation of LPC (18:1/0:0) and LPC (18:0/0:0) in MS group. Three years later, Rossi et al. [22] studied the sphingomyelin pathway since there is emerging evidence that increased CSF ceramides are linked to neuronal dysfunction [34,47,48]. The group using a lipidomics LC-MS/MS-based approach revealed that CSF sphingomyelins decreased in MS patients compared to OND patients. Some of their findings were that the levels of SM (d18:1/16:0), SM (d18:2/20:0), and SM (d18:1/14:0) decreased in the MS group.

In another lipidomics-based analysis, the authors of [20] analyzed the CSF lipidome and CSF fatty acids using both LC-MS/MS and GC-FID platforms. Characteristically, TG (64:10), 5-beta-dihydrotestosterone, a-linolenic acid, and arachidic acid were statistically increased in MS patients vs. control group, whereas TG (58:3), TG (52:2), and cholest-5-en-3alpha-ol followed the opposite trend in the same group comparison. The importance of fatty acids, glycerolipids, and sphingolipids analysis was also highlighted when the CSF and plasma lipid profiles of OND patients were compared to PPMS and RRMS ones using the UHPLC-TOFMS platform [21]. Sphingolipids species such as ceramides and hexosylceramide were also found statistically altered on a targeted LC-MS/MS sphingolipidomics analysis of brain tissue samples obtained from controls and OND and MS cohorts [34].

#### 2.2.2. Other Biological Fluids (Plasma, Serum, Urine, Tears)

A plethora of studies have investigated whether it is possible to differentiate MS patients, and the different disease forms, from healthy controls or OND or other immune-mediated CNS disease patients using other peripheral CNS biofluids, such as plasma, serum, urine, and tears. Again, both untargeted and targeted approaches, or their combination, were followed using hyphenated chromatographic techniques coupled with MS spectrometry or NMR spectroscopy. Dickens et al. [11] investigated whether a serum NMR metabolic profile was able to distinguish different forms of MS, namely RRMS, SPMS, and PPMS from healthy controls. Statistical analysis revealed significant differentiation between RRMS and SPMS groups. More specifically, fatty acids, phosphocholine, an N-acetyl species, and glucose were decreased, whereas other fatty acids and b-hydroxybutyrate were increased. Significant lipid alterations were also observed when disease groups were compared to the controls. The plasma metabolic profile was also able to separate MS patients from healthy controls in a large cohort study [36]. Statistical analysis indicated the alteration of eight metabolites in total, such as 3-OH-butyrate, acetone, alanine and choline with their levels increased in MS patients, as well as glucose, 5-OH-tryptophan, and tryptophan (decreased in MS). These findings suggest that metabolic perturbation occurs in tryptophan and energy metabolism.

Attention has also been given to the urine metabolic profile of MS patients due to the advantages it offers, including rapid and easy collection, quick availability, and noninvasive sampling. The metabolomics urine profile from healthy controls, MS, and NMO-SD patients measured by NMR revealed statistically significant differences among the three tested groups [12]. Some of the group’s findings were that isovalerate, 3-hydroxybutyrate, acetone, succinate, creatine, trimethylamine n-oxide, l-phenylalanine, and hippurate were found to be increased in the MS group compared to controls. In contrast, lactate, l-alanine, acetate, citrate, trimethylamine, choline, glycine, and xanthine were among the metabolites what were decreased. These findings suggest that branched amino acid metabolism, amino acid and derivatives metabolism, the TCA cycle, and glycine, serine, and threonine metabolism were most affected.

Besides NMR, hyphenated techniques (LC-MS, GC-MS) were also employed to exploit the presence of progressive MS based on the metabolic and/or lipid profile of patients. Stoessel et al. [15] demonstrated that specific plasma metabolic profiles could be linked with the diagnosis of PPMS. The group reported a panel of biomarkers with the potential to facilitate PPMS diagnosis. This panel exhibits a decreased concentration in plasma compared to healthy controls (HC), relapsing-remitting MS (RRMS), and Parkinson’s disease (PD) samples. Moreover, the group elucidated a dysregulation in pathways of glycerophospholipid and linoleic acid metabolisms in PPMS patients and advocate the importance of monitoring phosphatidylcholine (PC) moieties as markers of progression in PPMS. These conclusions are also supported in additional serum and plasma lipidomics-based studies [17,35,37,42] where mostly phosphatidylcholines (PCs) but also phosphatidylethanolamines (PEs), lysoPCs, and sphingomyelins (SMs) were found to be dysregulated in the MS cohorts.

In a similar approach to Stoessel’s group, Villoslada et al. [45] utilized an UHPLC-MS/MS method to discover and validate potential biomarkers for MS in two cohorts. The group performed untargeted metabolomics on cohort 1, whereas a targeted metabolomics approach was employed for cohort 2 for validation purposes. In cohort 2, a panel of metabolites and lipids were found to be significantly increased in the MS group. Among them, sphingomyelins (SMs) and lysophosphatidylethanolamines (lysoPEs) displayed a greater power in separating MS patients from controls. In addition, hydrocortisone, glutamic acid, and tryptophan were also found to be significantly altered, demonstrating that besides lipids, hormones and amino acids reflect MS pathogenesis.

The significant involvement of tryptophan and certain related metabolites, including indole lactate, indole propionate (gut microbiota derived), kynurenic acid, picolinic acid, quinolinic acid, and kynurenine was also highlighted in other studies using targeted metabolomics for the analysis of both adult and pediatric MS and CIS cohorts when compared to healthy controls [14,15,41]. Characteristically, Lim et al., [14] investigated the KP metabolic profile of MS patients. The samples investigated were serum from control RRMS, PPMS, and SPMS patients. By using a targeted metabolomics approach (UHPLC-fluorescence and GC-MS), they found increased levels of the kynurenine/tryptophan ratio, quinolinic acid, and 3-hydroxykynurenine in RRMS, PPMS, and SPMS compared to controls, while lower levels of nicotinamide adenine dinucleotide (NAD+) were observed in RRMS, PPMS, and SPMS groups. Moreover, besides the aromatic amino acid of tryptophan, other essential amino acids were also found to be highly related to the disease. Cicalini et al. [17] explored a more easily accessible biological fluid, tears, in addition to serum matrix, since they are considered as an intermediate fluid between the CSF and serum in terms of the relation to the components similar to other body fluids [49], by performing both untargeted lipidomics (tears) and targeted metabolomics (tears and serum) analyses using LC-MS/MS and Direct Infusion MS (DIMS). Among their findings is that several amino acids, including serine, asparagine, ornithine, arginine, glutamine, threonine, valine, as well as 15 PCs, six lysoPCs, 11 SMs, and certain acylcarnitines were found altered in the MS cohort compared to the controls.

In addition to the noteworthy changes observed in PC, PE, LysoPC, and SM lipids, it is imperative to acknowledge that alterations in various other lipid classes have also been identified, such as fatty acids (FAs), including monounsaturated fatty acids (MUFAs), polyunsaturated fatty acids (PUFAs), saturated fatty acids (SFAs), and very-long-chain fatty acid (VLCFA), as well as various ceramides [19,25,26,38,40]. Filippatou et al. [38] conducted a targeted LC-MS/MS lipidomics analysis monitoring 45 ceramides, with the aim to compare their levels between MS and healthy controls and to explore the prognostic power of the ceramide profile and its association with disability status. The group reported that certain ceramides, lactosylceramide and hexosylceramide, were statistically altered between controls and MS.

Furthermore, our group [16] has recently applied a serum targeted metabolomics LC-MS/MS analysis in order to identify distinct serum metabolomics patterns among patients with RRMS, CIS, and controls. OPLS-DA models demonstrated a clear separation between patients with CIS and controls, as well as patients with RMMS and the controls. Although initially we were not able to differentiate between CIS and RRMS, after performing hierarchical clustering analysis (HCA), three RRMS clusters were generated, and the OPLS-DA model was able to accurately discriminate between CIS and RRMS cluster 2 and 3 patients. Univariate analysis revealed 14 potential markers between CIS and RRMS cluster 2 and 3. Among them, betaine, cysteine, monoisoamylamine, and trimethylamine n-oxide (TMAO) were decreased in the serum of RRMS patients compared to CIS, while the opposite was observed for amino acids such as phenylalanine, serine, methionine, asparagine and threonine, purines, including xanthine and hypoxanthine, and the organic acid 2-hydroxyisobutyric acid (Table 2).

### 2.3. Metabolomics and Lipidomics in Animal Models of MS-like Disease and/or CNS Demyelination

Apart from human studies in MS, rodent studies in MS-like disease and/or CNS demyelination are reported. More specifically, the Experimental Autoimmune Encephalomyelitis (EAE) model is the most widely used animal model depicting aspects of MS pathophysiology as it is an experimental model of immune-mediated CNS demyelination (MS-like CNS disease) [50]. The cuprizone model is an experimental model of chemically-mediated CNS demyelination [51]. As summarized in Table 3, 6 articles were found [18,52,53,54,55] where metabolomics and lipidomic analyses were performed in samples of brain tissue and plasma in mouse models of CNS demyelinating disease. Among them, two articles studied plasma samples. Two articles studied only brain tissue (corpus callosum), and two articles conducted metabolomics analyses on plasma and urine samples.

Trepanier et al. [30] performed targeted lipidomics in cuprizone model brain tissue (corpus callosum) using GC-FID and LC-MS/MS. Amongst their findings, PC (34:1), PC (32:0), PC (36:4), and PC (38:6) were significantly increased in the cuprizone model compared to control samples, whereas oleic acid (18:1n-9), FA (20:0), FA (20:5n-3), FA (21:1n-9), and PC (36:1) were significantly decreased in the cuprizone model. In 2022, Zhao et al. [55] collected corpus callosum from controls and cuprizone model. They conducted targeted lipidomics using LC-MS/MS to investigate lipidomic alterations in the cuprizone model. They report eight lipids (cholesteryl esters, CE) that were significantly increased in the cuprizone model, while 112 lipids from different classes (PC, PE, TG, DG) were significantly decreased in the cuprizone model.

Lee, Hasan, Kwon, and Jung et al. [18] applied untargeted metabolomics and lipidomics to plasma from EAE and controls. Using an UHPLC-HR-MS/MS method, they found increased levels of glycerolipids, taurine-conjugated bile acids (BAs), and sphingolipids in the EAE model compared to controls. On the contrary, glycerophospholipids, lysoPCs, fatty acyls, and hydroxycholesterol were decreased in the EAE model. Poisson et al. [53] performed a metabolomics study of plasma in controls and EAE mice. After performing both UHPLC-HR-MS/MS and GC-MS methods, they found that 12-HΕΤΕ, 9-HODE, and 13-HODE levels were significantly increased in EAE, while levels of metabolites of ω-3 and ω-6 PUFAs significantly decreased in EAE. More specifically, regarding ω-3 PUFAs: Di-homo-gamma-linolenate (docosapentaenoic acid (22n-3)) and eicosapentaenoic acid were differentially decreased in EAE, whereas the ω-6 PUFAs dihomolinoleic acid, docosapentaenoic acid (22n-6), and arachidonic acid (AA) were also decreased in the EAE model.

### 2.4. Intervention Studies in Human MS and Animal MS-like Disease Using Metabolomics and Lipidomics

Apart from studies comparing the metabolomics and lipidomics profile from different stages of human patients and animal models of MS, intervention studies using omics in MS have also been reported. Although we include here recent intervention studies of MS, these were left out from the pathway analysis since our aim was to include studies that do not affect the metabolome and lipidome of MS human patients and animal models of MS.

Vaivade et al. [56] used autologous hematopoietic stem cell transplantation (AHSCT) since it has been showed to be highly effective in suppressing inflammation in RRMS patients. In this study, the effect of AHSCT on the metabolome and lipidome in peripheral blood from RRMS patients was investigated. The group showed that C16 ceramide, Cer (D18:2/16:0), and CerPE (d16:2(4E,6E)/22:0) were found to be statistically increased after AHSCT compared to prior to treatment. Furthermore, Troletti et al. [57] reported that treatment with the specialized pro-resolving lipid mediator (SPM) lipoxin A4 (LXA4) ameliorates clinical symptoms of experimental autoimmune encephalomyelitis (EAE). The group demonstrated that LXA4 affects the spinal cord lipidome by significantly reducing the levels of pro-inflammatory lipid mediators during EAE. Moreover, Stürner et al. [58] performed lipid mediator (LM) profiling by metabololipidomics in plasma samples from RRMS patients who took a standardized frankincense extract (SFE) for eight months and in healthy controls. Oral treatment with an SFE significantly reduces seven 5-lipoxygenase (5-LO)-derived LMs in RRMS patients during an eight-month treatment period. In addition, Siavoshi et al. [59] performed a longitudinal observational study where thirty-one RRMS patients were included. Using an untargeted metabolomics approach, the authors showed a significant alteration in circulating metabolome in RRMS patients undergoing ocrelizumab treatment, which is a highly efficacious therapy. More particularly, the group observed a significant reduction in metabolites involved in the lysophospholipid pathway, which was associated with patients’ improvement.

## 3. MS Metabolic Pathways Analysis

Based on the data drawn from the literature (Table 1, Table 2 and Table 3) Metabolic Pathway Analysis (MetPA), a feature in MetaboAnalyst 5.0, (https://www.metaboanalyst.ca/, accessed on 19 February 2024), was employed to scrutinize metabolic pathways, discerning significantly altered metabolites and lipids in various biological samples—brain, cerebrospinal fluid (CSF), plasma, serum, urine, and tears.

### 3.1. Metabolic Pathway Analysis among Human Studies

**Figure 1:**
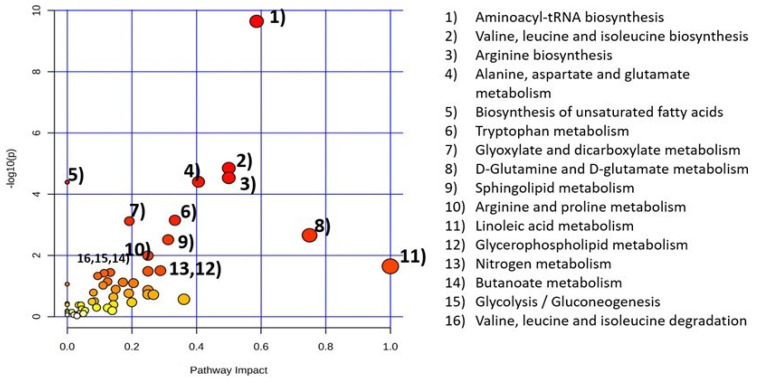
Metabolic pathway analysis in biological samples (brain, CSF, plasma, serum, urine, tears) of 31 human (RRMS, CIS, PPMS, SPMS) studies. The size of the circle corresponds to the pathway impact score (*x*-axis). Darker circle colors indicate more significant changes (*y*-axis) in metabolites in the corresponding pathway. Significant metabolic pathways (*p* < 0.05) are numbered in decreasing order (i.e., pathway 1 being most significant to pathway 16 being least significant).

After importing all the altered metabolites and lipids from 31 human studies on CNS demyelinating disease (RRMS, CIS, PPMS, SPMS) in brain, CSF, plasma, serum, urine, and tear samples, 16 metabolic pathways were found statistically significant as they had a *p*-value less than 0.05 (Figure 1). These include aminoacyl-tRNA biosynthesis; valine, leucine, and isoleucine biosynthesis; arginine biosynthesis; alanine, aspartate, and glutamate metabolism; biosynthesis of unsaturated fatty acids; tryptophan metabolism; glyoxylate and dicarboxylate metabolism; D-Glutamine and D-glutamate metabolism; sphingolipid metabolism; arginine and proline metabolism; linoleic acid metabolism; glycerophospholipid metabolism; nitrogen metabolism; butanoate metabolism; glycolysis/gluconeogenesis; and valine, leucine, and isoleucine degradation. Detailed information about pathway analysis results is given in Table S1.

### 3.2. Common Metabolic Pathways among Human Plasma and CSF Samples

**Figure 2:**
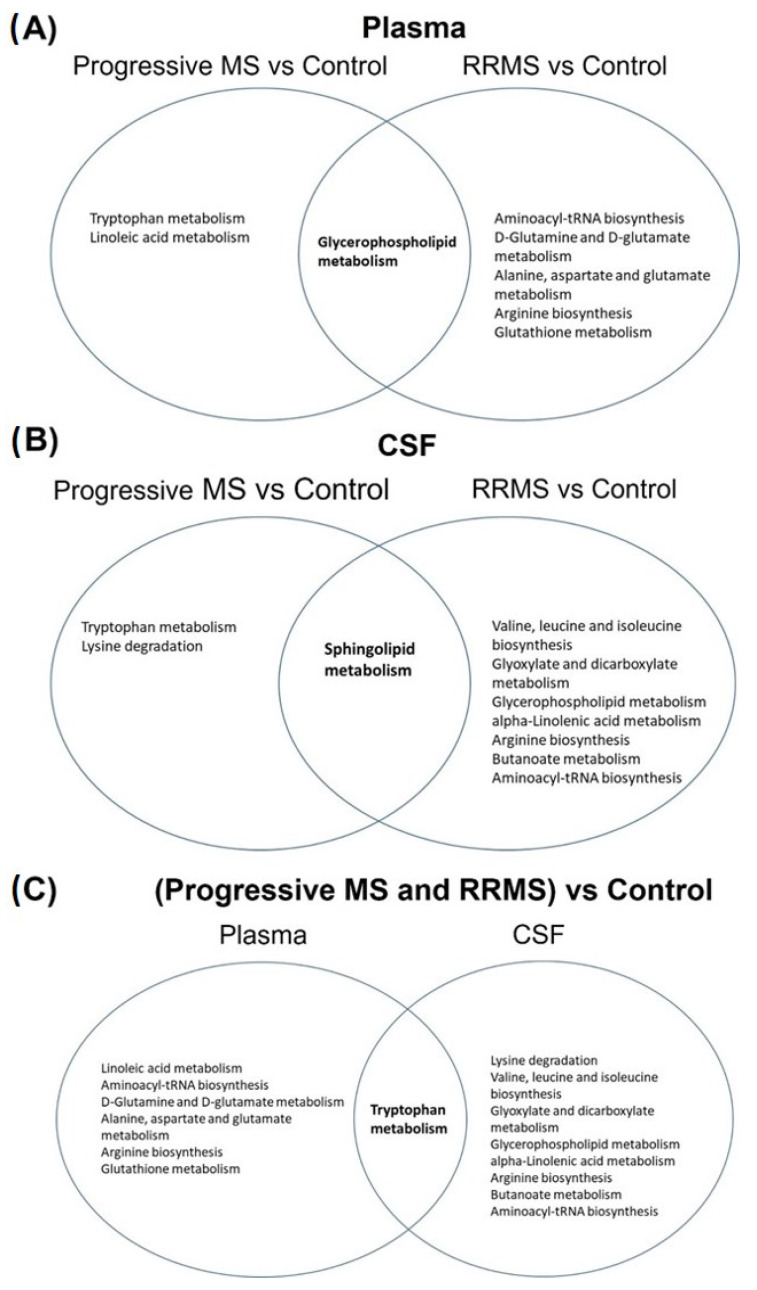
Dysregulated metabolic pathways in human samples, comparing different MS disease forms with healthy controls. (**A**) Intersection analysis of metabolic pathways dysregulated among progressive MS (PPMS, SPMS) and RRMS group human plasma samples. On the left are the significantly altered pathways in progressive MS vs. controls, on the right are all the significantly altered pathways in RRMS vs. controls, and the middle intersection is the common pathway (glycerophospholipid metabolism) among progressive and RMMS vs. control human plasma samples. (**B**) Intersection analysis of metabolic pathways among progressive MS (PPMS, SPMS) and RRMS group human CSF samples. On the left are all the altered pathways in progressive vs. control CSF samples, on the right are all the altered pathways in RRMS vs. control CSF samples, and the middle intersection is the common pathway (sphingolipid metabolism) among progressive and RMMS vs. control human CSF samples. (**C**) Intersection analysis of metabolic pathways among human plasma and CSF samples. On the left are all the altered pathways in combined progressive and RRMS vs. control plasma samples, on the right are all the altered pathways in combined progressive and RRMS vs. control CSF samples, and the middle intersection is the altered pathway in common (tryptophan metabolism) among human plasma and CSF samples.

Moreover, we examined which altered metabolic pathways were in common among the different matrices (i.e., plasma versus CSF) in RRMS and progressive MS (PPMS, SPMS) patients. For plasma, after importing all the altered metabolites in plasma samples from human RRMS, compared to controls, six metabolic pathways had a *p*-value lower than 0.05 (Table S2). When we imported all the altered metabolites in plasma samples from the human progressive MS (PPMS, SPMS), compared to controls, three metabolic pathways had a *p*-value less than 0.05 and an impact value greater than 0 (Table S3). One pathway, glycerophospholipid metabolism was common in plasma samples when we compared between RRMS versus controls and PPMS/SPMS versus controls (Figure 2A), which indicated that the metabolic profiles of RRMS and PPMS/SPMS share common pathological mechanisms.

Similarly, all the altered metabolites and lipids from CSF were analyzed among RRMS and PPMS/SPMS versus controls groups (Tables S4 and S5). One pathway, the sphingolipid metabolism pathway, was commonly shared in the studies of RRMS vs. controls and PPMS/SPMS vs. controls in CSF (Figure 2B).

We then used the combined (RRMS + PPMS/SPMS) altered metabolites and lipids found in the human plasma and CSF samples so as to reveal important pathways in common between these two sample matrices. It was shown that the tryptophan metabolism pathway was shared between the human plasma and CSF samples (Figure 2C).

### 3.3. Significantly Altered Metabolic Pathways among Mouse Models of CNS Demyelination

**Figure 3:**
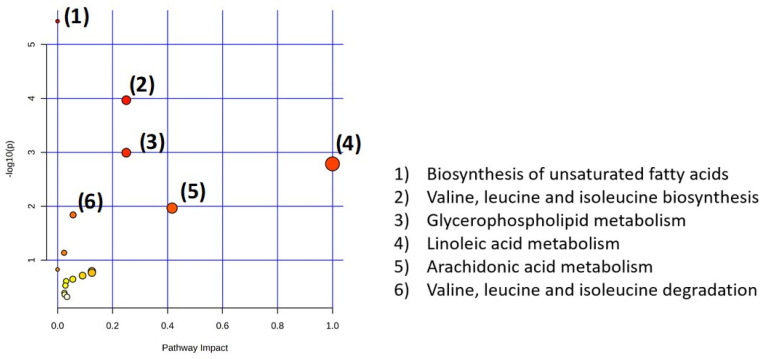
Metabolic pathway analysis in biological samples (brain and plasma) of 6 mouse models of CNS demyelination studies. The size of the circle corresponds to the pathway impact score (*x*-axis). Darker circle colors indicate more significant changes (*y*-axis) in metabolites in the corresponding pathway. Significant metabolic pathways (*p* < 0.05) are numbered in decreasing order (i.e., pathway 1 being most significant to pathway 6 being least significant).

For all the altered metabolites and lipid from the six mouse studies of different models of CNS demyelination (cuprizone, EAE) in brain, plasma and urine samples, when comparing to the control group, six metabolic pathways were found with a *p*-value less than 0.05 (Figure 3), including biosynthesis of unsaturated fatty acids; valine, leucine, and isoleucine biosynthesis; glycerophospholipid metabolism; linoleic acid metabolism; arachidonic acid metabolism; and valine, leucine, and isoleucine degradation.

## 4. Main Metabolic and Lipids Pathways with Respect to MS

Taking into account the findings from the above analysis in Section 3.1 and Section 3.3, five biological/lipid pathways were common between mouse models of CNS demyelination and human MS studies (all biofluids). These were biosynthesis of unsaturated fatty acids, valine, leucine and isoleucine biosynthesis, glycerophospholipid metabolism, linoleic acid metabolism, valine, leucine and isoleucine degradation. In the following section a description of these significantly altered metabolic pathways is given.

### 4.1. Biosynthesis of Unsaturated Fatty Acids

There are reports in the literature suggesting that unsaturated fatty acids (FAs) play a role in MS as early as 1973 [60] and 1982 [61]. Also, existing evidence indicates that the management of MS may be regulated, at least in part, through a properly balanced diet. The main concept is by eliminating the supply of animal fats and replacing with fats of plant origin, which contain omega-3 polyunsaturated fatty acids (PUFAs) [62]. However, the efficacy of PUFA supplementation on delaying MS deterioration is ambiguous according to an umbrella review from Tredinnick et al. [63]. This is corroborated another meta-analysis by Parks et al. [64], where PUFA administration could not halt disability in people with MS.

Nevertheless, there is a very recent report from Kim et al. [65], who studied the synergistic effect of omega-3 eicosanoids in T-cell activity in the EAE experimental model. The group found that docosahexaenoylethanolamide (DHEA), another dietary component, ameliorates disease severity in the relapse-remitting EAE (RR-EAE) model and may, therefore, serve as a complementary therapeutic scheme to existing MS medication. Moreover, a study by Poggioli et al. [66] demonstrated that omega-3 PUFA supplementation could attenuate disease deterioration in autoimmune diseases, like type 1 diabetes (T1D) and multiple sclerosis (MS). The beneficial role of omega-3, especially eicosapentaenoic acid (EPA) and docosahexaenoic acid (DHA), may stem from their anti-inflammatory properties on the cytokine release profile.

### 4.2. Valine, Leucine, and Isoleucine Biosynthesis

Singh et al. [54] studied the urine and the plasma profile in a chronic EAE model by global untargeted metabolomics. Eight common metabolites were found when the urinary and plasma biomarkers were combined, which indicates a dysregulation of phenylalanine metabolism and valine, leucine, and isoleucine biosynthesis pathways, which are involved in neurological diseases. Griffin et al. [67] investigated the NMR metabolic profiling of urine to detect inflammatory CNS lesions occurring in brains of rats. Statistical pattern recognition was used to detect inflammatory CNS lesions induced by microinjection of a recombinant adenovirus into the brains of Wistar rats. Biomarkers that account for group differentiation (control vs. adenovirus) are involved in the leucine, isoleucine, and valine biosynthesis pathway and myo-inositol.

### 4.3. Glycerophospholipid Metabolism

Ferreira et al. [37] demonstrated that the lipidomic profile may differentiate MS and healthy controls. More specifically, the group showed that certain phospholipids, namely PC (34:3), PC (36:6), PE (40:10), and PC (38:1) could be potential biomarkers for MS. In addition, Pousinis et al., in a previous study by the author of this publication [68], using an untargeted lipidomics UPLC-Q-TOF MS/MS approach, demonstrated that the lipid profile was able to separate not only controls from MS cases but also PPMS from SPMS with the use of NAWM human post-mortem brain tissue, with good prediction accuracy. Also, glycerophospholipid metabolism, glycerophosphatidyl inositol (GPI) anchor synthesis, and linoleic acid metabolism were the lipid pathways that were significantly altered between PPMS and SPMS. In addition, the group of Momchilova et al. [40] reported that the glycerophospholipid content was differentiated in erythrocytes and plasma between MS patients and healthy controls. In another study, Dong et al. [69] reported that oxidized phosphatidylcholines (OxPCs) found in MS lesions accounted for disease progression in MS.

### 4.4. Linoleic Acid Metabolism

Arshad et al. [70] studied the association of delta-6-desaturase expression (D6D) with MS. D6D drives the production of PUFAs in the body and maintains lipid homeostasis. The increased levels of D6D in MS serum show the important role that D6D has in inflammatory diseases such as MS. The authors suggested that ω6 and ω3 pathways are the main factors involved in the biosynthesis of PUFAs and D6D is the main enzyme that desaturates linoleic acid and alpha-linolenic acid.

### 4.5. Valine, Leucine, and Isoleucine Degradation

Hao et al. [71] investigated which genes are involved in MS pathogenesis. The group discovered that the highly expressed *CYBRD1* gene may be associated with “valine leucine and isoleucine degradation,” according to gene set enrichment analysis (GSEA).

## 5. Conclusions

In this review, we presented a summary of the latest metabolomics and lipidomics studies that were performed in biofluids of MS patients and mouse models of CNS demyelination. The altered pathways that were reported in these metabolomics and lipidomics studies were analyzed and five significantly altered metabolic pathways were common in MS patients and in experimental models of CNS demyelination. These pathways are biosynthesis of unsaturated fatty acids; valine, leucine, and isoleucine biosynthesis; glycerophospholipid metabolism; linoleic acid metabolism; and valine, leucine, and isoleucine degradation. The data indicate common alterations in terms of metabolic pathways between MS and experimental CNS demyelinating disease. Since obtaining human biofluids is limited by good clinical practice guidelines (e.g., limitations to obtain CSF from patients outside of the good clinical practice—patients with demyelinating disease typically undergo lumbar puncture for diagnostic purposes with no indication to undergo repeat lumbar puncture thereafter, unless indicated for other clinical purposes), in vivo experimental research can be considered as a useful alternative as it enables longitudinal sampling, harvesting samples from different compartments, in order to generate knowledge that may be complementary to clinical research in a translational setting.

We need to stress that our review of the literature studies comes with limitations. The coverage of metabolites and lipids presented a great diversity among studies due to different analytical platforms used (e.g., LC-MS, GC-MS, NMR) combined with different analytical methods and sampling methods employed in the laboratories under study. More importantly, it is commonly accepted within the metabolomics and lipidomics community that metabolomics and lipidomics results are challenging to reproduce even from the same human cohorts. Additionally, patients, and especially controls, may suffer from other diseases too, while MS-specific immunotherapy, commodities, and concomitant medications may arise as confounding factors. Furthermore, since metabolomics is defined as the systematic study of the unique chemical fingerprints that specific cellular processes leave behind, the study of their small-molecule metabolite profiles [72], we believe that the revealed metabolomics disturbances reflect the response of disease process. In addition to that, there is no indication in the publications under study as to whether the experimental findings in the metabolomics and lipidomics studies are the cause or the effect of the MS process.

To conclude, in this review, a summary and analysis of current metabolomics and lipidomics data were presented, covering both human and animal studies. This highlights the important contribution of experimental models in the MS research field as it may reproduce and provide proof-of-concept evidence for novel potential disease biomarkers and biological pathways. This holistic approach is expected to support early diagnosis of MS, to enhance monitoring of the disease, and to aid the discovery of MS potential therapeutic targets.

## Figures and Tables

**Table 1:** Summary of metabolomics and lipidomics studies (n = 11) carried out in cerebrospinal fluid (CSF) and brain tissue samples of PPMS, SPMS, and/or RRMS patients and controls.

Study Population	Metabolomics Approach	Analytical Platform	Potential Biomarkers in Case Group Compared with Control Group		Reference
			Increased levels	Decreased levels	
Control (n = 10) RRMS (n = 30) SPMS (n = 16)	Untargeted metabolomics (CSF)	UHPLC-HR-MS	SPMS vs. RRMS (in SPMS group) Ketoleucine, Trigonelline, Guanosine, N-Acetylphenylalanine, Isoleucine/Leucine, Phenylalanine, O-Succinyl-homoserine, N, Acetylleucine, Valine, Tyrosine, Phenylacetate, 1-Methyladenosine Cyclic AMP, 3,4-Dihydroxyphenylglycol, N6-(delta2-isopentenyl)-adenine, Glutamine, Methionine, Uridine, Kynurenine, 4-Acetamidobutanoate, Kynurenate, Urate, N-Acetylserotonin, 5-Hydroxytryptophan, Ethylmalonate, Glutarylcarnitine, Thymine, Pipecolate, 3-Methoxytyrosine, Biliverdin SPMS vs. Controls (in SPMS group) Ketoleucine, Guanosine, N-Acetylphenylalanine, N-Acetylleucine, 4-Hydroxybenzoate, Phenylacetate, Uridine, Trigonelline, Homogentisate N-Acetylphenylalanine, Xanthosine, O-Succinyl-homoserine, N6- (delta2-isopentenyl)-adenine, N-Acetyltryptophan, N6- (delta2-isopentenyl)-adenine, Pipecolate, 1-Methyladenosine, 4-Acetamidobutanoate, 4-Pyridoxate, Indole-3-acetate, Citrulline, 5-Hydroxytryptophan, N-Acetylserotonin, Kynurenate	SPMS vs. RRMS (in SPMS group) 4-Guanidinobutanoate, 3-Hydroxymethylglutarate, 5, Hydroxyindoleacetate, Deoxyuridine, 3-Methoxytyramine, Caffeine SPMS vs. Controls (in SPMS group) Carnitine, N-Acetylneuraminate, Deoxycarnitine, Indoxyl sulfate, Deoxyuridine, 5-Hydroxyindoleacetate, 3-Methoxytyramine, Caffeine	[46]
Control (n = 10) RRMS (n = 30) SPMS (n= 16)	Untargeted Metabolomics (CSF)	UHPLC-HR-MS	20β-dihydrocortisol (20β-DHF), Indolepyruvate (SPMS vs. RRMS)	-	[13]
Cohort 1 OND (n = 20) iOND * (n = 13) MS (n = 38) 1. RRMS (n = 24) -RRMS-remission (n = 16) -RRMS-relapse (n = 8) 2. PPMS (n = 5) 3. SPMS (n = 9) Cohort 2 RRMS (n = 48)	Targeted metabolomics (CSF)	LC-MS/MS and HPLC-FLD	RRMS relapse vs. OND (in RRMS group) Quinolinic acid, Quinolinic acid/Kynurenic acid and Quinolinic acid/Kynurenic acid ratios PPMS vs. OND (in PPMS group) Tryptophan, Kynurenic acid, Quinolinic acid	SPMS vs. OND (in SPMS group) Tryptophan, Kynurenic acid	[9]
Control (n = 17) (NMOSD, neuromyelitis optica spectrum disorder) (n = 57) MS (n = 50)	Untargeted metabolomics (CSF)	NMR	MS vs. control (in MS group) 2-hydroxybutyrate, Acetone, Formate, Pyroglutamate NMOSD vs. control (in NMOSD group) 2-hydroxybutyrate, Acetone, Formate, Pyroglutamate, Lactate	MS vs. Control (in MS group) Glucose, Citrate, Acetate NMOSD vs. Control (in NMOSD group) Acetate, Glucose	[32]
OND (n = 12) RRMS (n = 13)	Untargeted lipidomics, Targeted metabolomics (CSF)	MALDI-TOF LC-MS/MS	RRMS vs. OND (in RRMS group) LPC (18:1(9Z)/0:0), LPC (18:0/0:0), LPI (16:0/0:0), *m/z* 734.5 (PC), *m/z* 969.6 (PI) Glutamate	*m/z* 673.4 (PA)	[23]
Control (n = 19) MS (n = 19)	Untargeted metabolomics and lipidomics (CSF)	NMR	-	MS vs. Control (in MS group) Acetone, Choline, Urea, 1,3-dimethylurate, Creatinine, Isoleucine, Myo-inositol, Leucine, 3-OH-butyrate, Saturated and monounsaturated acyl groups of ω–9, ω–7, ω–6, ω–3, FA, TG, 1,3-DG, 1-MG, glycerol group in 1-MG (MS)	[24]
OND (n = 17) MS (n = 20)	Untargeted lipidomics (CSF)	LC-MS/MS	MS vs. OND (in MS group) PC (36:3), PC (32:2)	MS vs. OND (in MS group) PC (28:0), PC (28:1), PC (36:1), PC (36:8), PC (37:6), PC (35:4), LPC (18:1), LPC (20:4) SM (d18:1/16:0), SM (d18:2/20:0), SM (d18:1/24:1(15Z)), SM (d18:2/22:1), SM (d18:1/14:0), SM (d18:0/16:1(9Z) (OH)), SM (d18:1/13:0)	[22]
OIND (n = 8) IIH (idiopathic intracranial hypertension) (n = 14) Total MS cases (n = 13) PPMS (n = 2) RRMS (n = 11)	Untargeted lipidomics (CSF and plasma)	UHPLC-TOFMS	Fatty acids, Sphingolipids (CSF); Glycerolipids, Fatty acids (plasma)	-	[21]
Control/non-MS (n = 54) MS (n = 53) (RRMS)	Untargeted lipidomics, Targeted lipidomics (fatty acids) (CSF)	UPLC-MS/MS GC-FID	MS vs. Control (in MS group) TG (64:10), 5beta-dihydrotestosterone, 12-methyl-10-oxo-tridecanoic acid, [FA (18:3n3) (a-linolenic acid (ALA)], [FA (20:0) (arachidic acid)] (MS)	MS vs. Control (in MS group) TG (52:3), TG (58:3), TG (52:2), cholest-5-en-3alpha-ol (MS)	[20]
Control (n = 34) MS cases total (n = 24) ▪ RRMS (n = 22) ▪ PPMS (n = 2) Guillain–Barré syndrome (GBS) (n = 19)	Shotgun lipidomics (CSF)	DI-HRMS	MS vs. Control (in MS group) PA (38:4), PA (40:6), LPE (16:1), LPE (16:0), LPE (18:2), HexCer (34:1:2), HexCer (36:1:2), HexCer (38:2:2), HexCer (38:1:2), HexCer (40:1:2), HexCer (41:2:2), HexCer (41:1:2), HexCer (42:3:2), HexCer (41:1:3), GM3 (36:1:2), Cer (38:1:2), Cer (40:1:2), DG (32:1), DG (32:0), DG (34:1), TG (44:1), TG (44:0), TG (46:2), TG (46:1), TG (46:0), TG (48:3), TG (48:0), TG (50:0), TG (54:1), PS (36:1), PS (40:4), PG (44:12), PA (36:1)	MS vs. Control (in MS group) LPE (18:1), CE (22:6)	[33]
Control; normal CNS (n = 3) OND (n = 15) MS (n = 13); Chronic Active MS (Ac-MS) (n = 4) Inactive MS (n = 9)	Targeted sphingolipidomics (brain tissue; normal-appearing white matter, NAWM)	LC-MS/MS	In-MS lesions in comparison to Ac-MS lesions; C1P (4.2-fold increase), HexCer (4.0-fold increase), Sphingoid (1.9-fold increase) content	In-MS lesions in comparison to Ac-MS lesions; Cer (4.4-fold decrease), dhCer (3.6-fold decrease), SM (1.6-fold decrease) content	[34]

* inflammatory OND (iOND).

**Table 2:** Summary of metabolomics studies (n = 21) carried out in peripheral CNS biofluids (plasma, serum, urine, and tears) in CIS, PPMS, SPMS, and/or RRMS disease patients, also compared to other immune-mediated CNS disease and/or healthy controls.

Study Population	Metabolomics Approach	Analytical Platform	Potential Biomarkers in Case Group Compared with Control Group		Ref.
			Increased levels	Decreased levels	
Control (n = 24) RRMS (n = 26) PPMS (n = 13) SPMS (n = 58)	Untargeted Metabolomics (serum)	NMR	SPMS vs. RRMS (in SPMS group) Fatty acids, b- hydoxybutyrate RRMS, SPMS vs. Controls (in MS groups) Fatty acids PPMS vs. Controls (in PPMS group) Lactate, N-acetyl species, Fatty acids	SPMS vs. RRMS (in SPMS group) Fatty acids, Phosphocholine, N-acetyl species, Glucose MS vs. Control (in MS group) Glucose, Phosphocholine + Fatty acids, Lactate (RRMS, SPMS)	[11]
RRMS (n = 34) Aquaporin-4 (AQP4)- antibody (Ab) seropositive neuromyelitis optica spectrum disorder (NMOSD) (n = 54) Myelin oligodendrocyte glycoprotein (MOG)-Ab disease (n = 20)	Untargeted metabolomics (plasma)	NMR	In AQP4-Ab(+) Concentration of large LDL particles, Size of HDL particles, Glucose, Cholesterol concentration in large HDL, Large LDL particles; In RRMS Lysine/Creatinine/Creatine, Histidine In MOG-Ab(+) Formate, Leucine	In AQP4-Ab Small HDL particles, Phosphocholine/lipoprotein Scyllo-inositol In RRMS Large HDL particles, Lactate, Unsaturated lipids, Alanine In MOG-Ab Myo-inositol	[39]
Control (n = 88) Cases total (n = 73) -RRMS (n = 61) -Progressive (n = 12)	Untargeted metabolomics (plasma)	NMR	MS vs. Control (in MS group) 3-OH-butyrate, Acetoacetate, Acetone, Alanine, Choline	MS vs. Control (in MS group) Glucose, 5-OH-tryptophan, Tryptophan (MS)	[36]
Control (n = 7) Aquaporin-4 (AQP4)-seropositive NMO-SD) (n = 9) RRMS (n = 8)	Untargeted metabolomics (urine)	NMR	MS vs. Controls (in MS group) 2-hydroxyisovalerate, isovalerate, 3-hydroxybutyrate, 3-hydroxyisovalerate, Acetone, Acetoacetae, Oxaloacetate, Succinate, Creatine, Malonate, Trimethylamine n-oxide, l-Phenylalanine, Phenylacetylglycine, Hippurate NMO-SD vs. Controls (in NMO group) Acetate, N-Acetylglutamine, Acetoacetae Oxaloacetate, Succinate, Trimethylamine Creatine, Malonate, Trimethylamine n-oxide, L-Phenylalanine, Phenylacetylglycine, Hippurate	MS vs. Controls (in MS group) 3-hydroxyisobutyrate, Propylene glycol, Methylmalonate, Lactate, L-Alanine, Acetate, N-acetylglutamine, Citrate, Trimethylamine, Choline, Glycine, Xanthine MS vs. Controls (in MS group) 3-hydroxybutyrate, Methylmalonate 3-hydroxyisovalerate, Lactate, L-Alanine, Acetone, Citrate, Creatinine, Choline, Glycine, Xanthine	[12]
Control (n = 33) PPMS (n = 33) RRMS (n = 10)	Untargeted metabolomics (plasma)	UHPLC-HR-MS/MS	PPMS vs. Controls (in PPMS group) Tiglylcarnitine (PPMS vs. RRMS); Gamma-Linolenic acid, (L)-tryptophan, LysoPC (20:0) (RRMS)	PPMS vs. RRMS (in group) Citrulline, Creatinine, (L)-tryptophan, LysoPE (18:1), LysoPE (18:2), LysoPE (22:4), LysoPC (P-16:0), LysoPC (P-18:0), LysoPC (P-18:1), PC (44:12), LysoPC (20:1), LysoPC (20:0), PE (36:5), PC (35:5), PC (18:1/18:1), PC (18:0/18:3), Tiglylcarnitine, 2(R)-HOT, GPC (14:0), Gamma-Linolenic acid (PPMS); LysoPE (18:2), LysoPC (20:0)	[15]
Control (n = 33) Cases total (n = 32) RRMS (n = 28) Progressive (n = 4)	Untargeted metabolomics (plasma)	GC-MS	L-Asparagine, L-Ornithine, L-Glutamine, L-Glutamate (MS)	Phosphate, Fructose, Myo-Inositol, Pyroglutamate, Threonate, L-Leucine (MS)	[43]
Cohort 1 (prospective) Control (n = 41) Cases total (n = 61) CIS (n = 16) RRMS (n = 33) PPMS (n = 4) SPMS (n = 8) Cohort 2 (validation) Control (n = 74) Cases total (n = 238) CIS (n = 39) RRMS (n = 192) PPMS (n = 4) SPMS (n = 6)	Cohort 1 (prospective) Untargeted metabolomics (serum) Cohort 2 (retrospective/validation) Targeted metabolomics (serum)	Cohort 1 UHPLC-MS Cohort 2 UHPLC-MS/MS	Cohort 2 (validation) PC (15:0/22:6), Arachidonic acid, 13-hydroxyoctadecadienoic acid (13-HODE), lysoPC (20:0/0:0), lysoPC (20:1/0:0), lysoPC (22:5/0:0), PC (17:0/0:0), Hydrocortisone, Glutamic acid, Tryptophan, Eicosapentaenoic acid (Timnodonic acid), 13S-Hydroxyoctadecadienoic acid, lysoPC (20:5/0:0), lysoPE (20:5/0:0) (MS)	-	[45]
Control (n = 13) MS (n = 12)	Untargeted and targeted metabolomics (serum)	GCXGC-TOF MS LC-MS/MS	Pyroglutamate, Laurate, Acylcarnitine C14:1), PC ae 42:5, PC ae 40:5, N-Methylmaleimide (MS)	-	[10]
Pediatric Control (n = 67) MS/clinically isolated syndrome (CIS) (n = 69)	Untargeted and targeted metabolomics (serum)	UHPLC-MS/MS	Increased levels of tryptophan and indole lactate associated with decreased risk of MS	-	[41]
Control (n = 49) RRMS (n = 50) PPMS (n = 17) SPMS (n = 20)	Targeted metabolomics (serum)	UHPLC-fluorescence GC-MS	Kynurenine/Tryptophan (K/T) ratio, Quinolinic acid (QA), 3-hydroxykynrenine (3-HK) (RRMS, PPMS, SPMS); Kynurenic acid (KA), Picolinic acid (PA) (RRMS); QA/KA (PPMS, SPMS)	Kynurenic acid (KA), Picolinic acid (PA) (PPMS, SPMS); Nicotinamide adenine dinucleotide (NAD^+^) (RRMS, PPMS, SPMS)	[14]
Control (n = 13) RRMS (n = 13)	Targeted metabolomics (plasma)	LC-MS/MS	-	Methionine (RRMS)	[44]
Controls (n = 30) RRMS (n = 24)	Untargeted lipidomics (serum)	UHPLC-MS/MS		PC (34:3), PC (36:6), PE (40:10) and PC (38:1) (RRMS)	[37]
Controls (n = 8) PPMS-NP (PPMS with non-progressed disability (n = 10) PPMS-P (PPMS with progressed disability) (n = 9)	Untargeted lipidomics (plasma)	LC-MS/MS direct infusion shotgun lipidomics	Sphingomyelin-d18:1/14:0, Mono-hexosylceramide-d18:1/20:0 (PPMS vs. controls)	Lyso-phosphatidic acid-18:2 (LPA-18:2) (PPMS vs. controls)	[35]
73 monozygotic twin pairs with an MS diagnosis	Untargeted lipidomics (plasma)	Shotgun lipidomics	-	A significant reduction in ether phosphatidylcholines (PC O-), Ether phosphatidylethanolamines (PE O-) and phosphatidylcholines (PC) in MS co-twins. After correction for multiple comparison, PC O-16:1;0/20:3;0 remained as true discovery.	[42]
Tears Control (n = 21) MS (n = 12) Serum Control (n = 10) MS (n = 12)	Untargeted lipidomics (tears) Targeted metabolomics (tears and serum)	LC-MS/MS DIMS (Direct Infusion MS)	Ser, His, Asp; Acylcarnitines C5OH/C4DC, C10:1, C8:1 (tears, MS) Ser, Asp, Asn, Thr, Pro, Val, Ala, Orn, Leu/Ile/Pro-OH, Glu, Arg (serum, MS)	15 PCs, 6 lysoPCs, 11 SMs (tears, MS); Acylcarnitines C12, C14:1, C18:1OH (tears, MS) His, Tyr, Lys/Gln (serum, MS)	[17]
Control (n = 5) RRMS (n = 8)	Untargeted and targeted lipidomics (plasma)	MALDI-TOF/TOF GC-FID	(PG 42:0 or PI 36:2), PI 40:5, CL 72:8, CL 74:10, Σ MUFA, Σ PUFA, Total n-6 FAs (RRMS) Cardiolipin; Monounsaturated fatty acids, Polyunsaturated fatty acids,	Σ SFA (RRMS) Saturated fatty acids	[26]
Control (n = 55) RRMS (n = 100) PPMS (n = 19) SPMS (n = 24)	Targeted Lipidomics (plasma)	FIA-MS/MS	VLCFA-PtdEtn, GTAs (RRMS > 13 y); PtdEtn 16:0/28:0 (SPMS); PlsEtn 16:0/22:6 (SPMS and RRMS > 13 y)	GTAs (SPMS)	[25]
Control (n = 301) MS (n = 102)	Targeted lipidomics (serum)	LC-MS/MS	LacCerC24:1, C16Sphinganin (MS) LacCer = lactosylceramide	GluCerC16, HETE15S, LPA20:4, biopterin, OEA, PEA GluCer = glucosylceramide, eicosanoid, endocannabinoid	[19]
Control (n = 18) RRMS (n = 18)	Fatty acid profile Ceramide content (plasma)	GC-MS HPLC-UV	C 16:0, C 18:0, Ceramide (RRMS)	C 18:2, C 22:4, C 22:6 (RRMS)	[40]
Controls (n = 68) RRMS (n = 151) PMS (n = 100) (Progressive MS)	Targeted lipidomics (serum)	LC-MS/MS	Cer16:0, Cer22:1, Hex-Cer24:1, Lac-Cer24:1, Lac-Cer22:0, DH-Cer20:0, DH-Cer24:0 (MS)	Hex-Cer16:1, Lac-Cer20:1, DH-HexCer26:0 (MS)	[38]
Controls (n = 11) CIS (n = 11) RRMS (n = 37)	metabolomics (serum)	LC-MS/MS	Phenylalanine, Serine, Methionine, Aspartic acid, Asparagine, Glutamic acid, Threonine, Xanthine, Hypoxanthine, 2-hydroxyisobutyric acid.	Betaine, Cysteine, Monoisoamylamine, Trimethylamine n-oxide (TMAO) (in RRMS group)	[16]

**Table 3:** Summary of metabolomics studies (n = 6) carried out on biofluids in rodent models of CNS demyelination.

Study Population	Metabolomics Approach	Analytical Platform	Potential Biomarkers in Case Group Compared with Control Group		Ref.
			Increased levels	Decreased levels	
Animal model: Control (n = 5) Cuprizone (n = 4)	Targeted lipidomics (brain tissue) (brain tissue: corpus callosum)	GC-FID, LC-MS/MS	PC (32:1), PC (34:1), PC (30:0), PC (32:0), PC (34:2), PC (36:4), PC (38:6) (cuprizone model)	Oleic acid (18:1n-9), FA (20:0), FA (20:5n-3), FA (21:1n-9), FA (22:1n-9), FA (24:1n-9), PC 36:1 (cuprizone model)	[30]
Animal model: Control (n = 5) Experimental autoimmune encephalomyelitis (EAE) (n = 5)	Untargeted metabolomics and lipidomics (plasma)	UHPLC-HR-MS/MS	Glycerolipids, taurine-conjugated bile acids (BAs), sphingolipids (EAE)	Glycerophospholipids, LysoPCs, fatty acyls, hydroxycholesterol (EAE)	[18]
Animal model: Control (n = 5) EAE (n = 5)	Untargeted metabolomics (urine and plasma)	GC-MS, UPLC-MS	-	3-methyl-2-oxovalerate, 4-methyl-2-oxopentanoate, allantoin, N-formylmethionine, p-cresol-sulfate, phenylacetylglycine, pseudouridine (EAE)	[54]
Animal model Control (n = 5) EAE (n = 5)	Untargeted metabolomics (plasma)	UHPLC-HR-MS/MS GC-MS	12-HΕΤΕ, 9-HODE, 13-HODE (EAE)	Metabolites of *ω*-3,*ω*-6 PUFAs (EAE) *ω*-3 PUFAs: Di-homo-gamma-linolenate, (docosapentaenoic acid (22n-3)), eicosapentaenoic acid; *ω*-6 PUFAs: dihomolinoleic acid, docosapentaenoic acid (22n-6), arachidonic acid	[53]
Animal model urine Control (n = 4) Chronic relapsing EAE (Cr-EAE) (n = 5) Plasma Control (n = 5) Cr-EAE (n = 5)	Untargeted metabolomics (plasma and urine)	NMR	Glucose, creatine, trimethylamine-N-oxide (TMAO) (urine, MS); glucose, choline (plasma, MS)	Fatty acids (plasma, MS)	[52]
Animal model Control (n = 10) Cuprizone (n = 10)	Targeted lipidomics (corpus callosum)	LC-MS/MS	8 lipids (cuprizone); CE	112 lipids (cuprizone); PC, PE, TG, DG	[55]

## Data Availability

The original contributions presented in the study are included in the article/Supplementary Materials, further inquiries can be directed to the corresponding author.

## Supplementary Materials

The following supporting information can be downloaded at: https://www.mdpi.com/article/10.3390/metabo14100545/s1. Table S1. Metabolic pathway analysis (MetPA) of studies in human biofluids; Table S2. Metabolic pathway analysis (MetPA) of studies in human plasma of RRMS patients versus healthy controls; Table S3. Metabolic pathway analysis (MetPA) of studies in human plasma of progressive MS (PPMS, SPMS) patients versus healthy controls; Table S4. Metabolic pathway analysis (MetPA) of studies in human CSF of RRMS patients versus healthy controls; Table S5. Metabolic pathway analysis (MetPA) of studies in human CSF of progressive MS (PPMS, SPMS) patients versus healthy controls; Table S6. Metabolic pathway analysis (MetPA) of studies in mouse models of CNS demyelinating disease in all biofluids and brain tissue.
