# Supplementary material for: Recent Advances in Metabolomics and Lipidomics Studies in Human and Animal Models of Multiple Sclerosis
Source: Metabolites. 2024 Oct 13;14(10):545. doi: 10.3390/metabo14100545 (PMC11509141; doi:10.3390/metabo14100545)
Supplement: Supplementary file 1 [file metabolites-14-00545-s001.zip › metabolites-3240306-supplementary.pdf]

SUPLEMENTARY INFO_Tables S1-S6.

Supplementary Table S1. Metabolic pathway analysis (MetPA) of studies in human biofluids (plasma, serum, CSF, urine, tears, brain tissue). 16 pathways (in bold) are significant (i.e. p<0.05). P values which are significant are highlighted in green.

Metabolic pathways	Total	Expected	Hits	Raw p	"-log10p"	Holm adjust	FDR	Impact
**Aminoacyl-tRNA biosynthesis**	48	2.4155	16	2.26E-10	9.6454	1.90E-08	1.90E-08	0.58617
**Valine, leucine and isoleucine biosynthesis**	8	0.40258	5	1.41E-05	4.8493	0.0011744	0.00059425	0.5
**Arginine biosynthesis**	14	0.70452	6	2.92E-05	4.5348	0.0023935	0.00068194	0.5
**Alanine, aspartate and glutamate metabolism**	28	1.409	8	3.94E-05	4.4045	0.0031911	0.00068194	0.40625
**Biosynthesis of unsaturated fatty acids**	36	1.8116	9	4.06E-05	4.3916	0.0032473	0.00068194	0
**Tryptophan metabolism**	41	2.0632	8	0.00070907	3.1493	0.056017	0.0090947	0.33332
**Glyoxylate and dicarboxylate metabolism**	32	1.6103	7	0.0007579	3.1204	0.059116	0.0090947	0.1923
**D-Glutamine and D-glutamate metabolism**	6	0.30194	3	0.0021985	2.6579	0.16928	0.023084	0.75
**Sphingolipid metabolism**	21	1.0568	5	0.0030599	2.5143	0.23255	0.028559	0.3125
**Arginine and proline metabolism**	38	1.9123	6	0.010161	1.9931	0.76205	0.08535	0.25
**Linoleic acid metabolism**	5	0.25161	2	0.022647	1.645	1	0.17294	1
**Glycerophospholipid metabolism**	36	1.8116	5	0.031372	1.5035	1	0.2124	0.28845
**Nitrogen metabolism**	6	0.30194	2	0.032871	1.4832	1	0.2124	0.25
**Butanoate metabolism**	15	0.75484	3	0.036016	1.4435	1	0.21359	0.13334
**Glycolysis / Gluconeogenesis**	26	1.3084	4	0.038141	1.4186	1	0.21359	0.11428
**Valine, leucine and isoleucine degradation**	40	2.0129	5	0.046837	1.3294	1	0.24589	0.09435
Ubiquinone and other terpenoid-quinone biosynthesis	9	0.4529	2	0.071534	1.1455	1	0.35194	0.125
Citrate cycle (TCA cycle)	20	1.0065	3	0.075416	1.1225	1	0.35194	0.17242
Glycine, serine and threonine metabolism	33	1.6606	4	0.080185	1.0959	1	0.3545	0.20513
Phenylalanine metabolism	10	0.50323	2	0.08657	1.0626	1	0.3636	0
Pyruvate metabolism	22	1.1071	3	0.094882	1.0228	1	0.37953	0.11112
Lysine degradation	25	1.2581	3	0.12746	0.89464	1	0.48665	0.15
alpha-Linolenic acid metabolism	13	0.65419	2	0.13628	0.86556	1	0.49773	0.25

Glutathione metabolism	28	1.409	3	0.16343	0.78668	1	0.57199	0.08109
Nicotinate and nicotinamide metabolism	15	0.75484	2	0.17217	0.76404	1	0.5785	0.19048
Phenylalanine, tyrosine and tryptophan biosynthesis	4	0.20129	1	0.18677	0.7287	1	0.59316	0.25
Histidine metabolism	16	0.80516	2	0.19066	0.71974	1	0.59316	0.26667
Synthesis and degradation of ketone bodies	5	0.25161	1	0.2278	0.64245	1	0.68339	0.14286
Arachidonic acid metabolism	36	1.8116	3	0.27032	0.56813	1	0.78299	0.36111
Pyrimidine metabolism	39	1.9626	3	0.31254	0.50509	1	0.87512	0.08475
Propanoate metabolism	23	1.1574	2	0.32365	0.48992	1	0.877	0.07692
Ascorbate and aldarate metabolism	8	0.40258	1	0.33901	0.46978	1	0.88991	0.2
Vitamin B6 metabolism	9	0.4529	1	0.37245	0.42893	1	0.94457	0
Biotin metabolism	10	0.50323	1	0.40421	0.39339	1	0.94457	0
Caffeine metabolism	10	0.50323	1	0.40421	0.39339	1	0.94457	0.14286
Purine metabolism	65	3.271	4	0.41582	0.38109	1	0.94457	0.03489
Phosphatidylinositol signaling system	28	1.409	2	0.41606	0.38085	1	0.94457	0.04256
Cysteine and methionine metabolism	33	1.6606	2	0.50195	0.29934	1	1	0.09091
Glycosylphosphatidylinositol (GPI)-anchor biosynthesis	14	0.70452	1	0.51615	0.28722	1	1	0.125
Glycerolipid metabolism	16	0.80516	1	0.56406	0.24867	1	1	0.04545
Pantothenate and CoA biosynthesis	19	0.95613	1	0.62728	0.20254	1	1	0.05556
Tyrosine metabolism	42	2.1135	2	0.63501	0.19722	1	1	0.13954
Selenocompound metabolism	20	1.0065	1	0.64627	0.18959	1	1	0
beta-Alanine metabolism	21	1.0568	1	0.6643	0.17764	1	1	0
Fatty acid biosynthesis	47	2.3652	2	0.69629	0.15721	1	1	0.01449
Galactose metabolism	27	1.3587	1	0.75493	0.12209	1	1	0
Porphyrin and chlorophyll metabolism	30	1.5097	1	0.79071	0.10198	1	1	0.03846
Inositol phosphate metabolism	30	1.5097	1	0.79071	0.10198	1	1	0.05
Amino sugar and nucleotide sugar metabolism	37	1.8619	1	0.85537	0.067847	1	1	0.02564
Fatty acid elongation	39	1.9626	1	0.8699	0.06053	1	1	0
Fatty acid degradation	39	1.9626	1	0.8699	0.06053	1	1	0.02041
Steroid hormone biosynthesis	85	4.2774	2	0.93682	0.028344	1	1	0.0303

Supplementary Table S2. Metabolic pathway analysis (MetPA) of studies in human plasma of RRMS patients versus healthy controls. 6 pathways (in bold) are significant (i.e. $p<0.05$). P values which are significant are highlighted in green.

Plasma_RRMS vs controls_pathways	Total	Expected	Hits	Raw p	"-log10p"	Holm adjust	FDR	Impact
**Aminoacyl-tRNA biosynthesis**	48	0.52645	6	6.14E-06	5.2117	0.0005159	0.0005159	0.20688
**D-Glutamine and D-glutamate metabolism**	6	0.065806	2	0.0016559	2.781	0.13744	0.069546	0.5
**Alanine, aspartate and glutamate metabolism**	28	0.3071	3	0.0030329	2.5181	0.2487	0.084922	0.21875
**Glycerophospholipid metabolism**	36	0.39484	3	0.0062593	2.2035	0.507	0.13145	0.17307
**Arginine biosynthesis**	14	0.15355	2	0.0095393	2.0205	0.76314	0.16026	0.125
**Glutathione metabolism**	28	0.3071	2	0.036209	1.4412	1	0.50693	0.05406
Nitrogen metabolism	6	0.065806	1	0.064129	1.1929	1	0.7109	0
Tryptophan metabolism	41	0.44968	2	0.072274	1.141	1	0.7109	0.15384
Ascorbate and aldarate metabolism	8	0.087742	1	0.084631	1.0725	1	0.7109	0.2
Valine, leucine and isoleucine biosynthesis	8	0.087742	1	0.084631	1.0725	1	0.7109	0.25
Glycosylphosphatidylinositol (GPI)-anchor biosynthesis	14	0.15355	1	0.14363	0.84276	1	1	0.125
Selenocompound metabolism	20	0.21935	1	0.19903	0.70107	1	1	0
Glycolysis / Gluconeogenesis	26	0.28516	1	0.25105	0.60023	1	1	0.02857
Galactose metabolism	27	0.29613	1	0.25941	0.58602	1	1	0
Phosphatidylinositol signaling system	28	0.3071	1	0.26767	0.57239	1	1	0.02128
Inositol phosphate metabolism	30	0.32903	1	0.28395	0.54676	1	1	0.05
Glyoxylate and dicarboxylate metabolism	32	0.35097	1	0.29988	0.52305	1	1	0
Glycine, serine and threonine metabolism	33	0.36194	1	0.30772	0.51184	1	1	0.02564
Cysteine and methionine metabolism	33	0.36194	1	0.30772	0.51184	1	1	0.0303
Biosynthesis of unsaturated fatty acids	36	0.39484	1	0.33075	0.4805	1	1	0
Amino sugar and nucleotide sugar metabolism	37	0.40581	1	0.33826	0.47074	1	1	0.02564

Arginine and proline metabolism	38	0.41677	1	0.3457	0.4613	1	1	0.05
Pyrimidine metabolism	39	0.42774	1	0.35306	0.45216	1	1	0.01695
Valine, leucine and isoleucine degradation	40	0.43871	1	0.36034	0.44329	1	1	0.01887
Purine metabolism	65	0.7129	1	0.51913	0.28473	1	1	0.01163

Supplementary Table S3. Metabolic pathway analysis (MetPA) of studies in human plasma of progressive MS (PPMS, SPMS) patients versus healthy controls. 3 pathways (in bold) are significant (i.e. p<0.05). P values which are significant are highlighted in green.

Plasma_Progressive MS vs controls_pathways	Total	Expected	Hits	Raw p	"-log10p"	Holm adjust	FDR	Impact
**Glycerophospholipid metabolism**	36	0.27871	5	3.58E-06	5.4459	0.00030088	0.00030088	0.28845
**Tryptophan metabolism**	41	0.31742	2	0.038107	1.419	1	1	0.15384
**Linoleic acid metabolism**	5	0.03871	1	0.038163	1.4184	1	1	0.25
Aminoacyl-tRNA biosynthesis	48	0.37161	2	0.050866	1.2936	1	1	0.06896
alpha-Linolenic acid metabolism	13	0.10065	1	0.096457	1.0157	1	1	0.125
Arginine biosynthesis	14	0.10839	1	0.10351	0.98501	1	1	0.0625
Glycosylphosphatidylinositol (GPI)-anchor biosynthesis	14	0.10839	1	0.10351	0.98501	1	1	0.125
Glycerolipid metabolism	16	0.12387	1	0.11747	0.93008	1	1	0.04545
Selenocompound metabolism	20	0.15484	1	0.14479	0.83927	1	1	0
Sphingolipid metabolism	21	0.16258	1	0.1515	0.8196	1	1	0.0625
Glycolysis / Gluconeogenesis	26	0.20129	1	0.18432	0.73444	1	1	0.02857
Phosphatidylinositol signaling system	28	0.21677	1	0.19711	0.70528	1	1	0.02128
Alanine, aspartate and glutamate metabolism	28	0.21677	1	0.19711	0.70528	1	1	0.03125
Glycine, serine and threonine metabolism	33	0.25548	1	0.22831	0.64147	1	1	0.02564
Biosynthesis of unsaturated fatty acids	36	0.27871	1	0.24649	0.6082	1	1	0
Arachidonic acid metabolism	36	0.27871	1	0.24649	0.6082	1	1	0.02778

Supplementary Table S4. Metabolic pathway analysis (MetPA) of studies in human CSF of RRMS patients versus healthy controls. 8 pathways (in bold) are significant (i.e. p<0.05). P values which are significant are highlighted in green.

CSF_RRMS vs controls; pathways	Total	Expected	Hits	Raw p	"-log10p"	Holm adjust	FDR	Impact
**Sphingolipid metabolism**	21	0.29806	3	0.0027996	2.5529	0.23517	0.21486	0.25
**Valine, leucine and isoleucine biosynthesis**	8	0.11355	2	0.0051158	2.2911	0.42461	0.21486	0.25
**Glyoxylate and dicarboxylate metabolism**	32	0.45419	3	0.0094331	2.0253	0.77351	0.21676	0.11538
**Glycerophospholipid metabolism**	36	0.51097	3	0.013088	1.8831	1	0.21676	0.15384
**alpha-Linolenic acid metabolism**	13	0.18452	2	0.013651	1.8648	1	0.21676	0.25
**Arginine biosynthesis**	14	0.19871	2	0.01579	1.8016	1	0.21676	0.125
**Butanoate metabolism**	15	0.2129	2	0.018063	1.7432	1	0.21676	0.13334
**Aminoacyl-tRNA biosynthesis**	48	0.68129	3	0.02839	1.5468	1	0.29809	0.10344
Glycolysis / Gluconeogenesis	26	0.36903	2	0.05089	1.2934	1	0.44436	0.05714
Glutathione metabolism	28	0.39742	2	0.05819	1.2352	1	0.44436	0.05406
Alanine, aspartate and glutamate metabolism	28	0.39742	2	0.05819	1.2352	1	0.44436	0.15625
Synthesis and degradation of ketone bodies	5	0.070968	1	0.069068	1.1607	1	0.44629	0.14286
Linoleic acid metabolism	5	0.070968	1	0.069068	1.1607	1	0.44629	0.25
D-Glutamine and D-glutamate metabolism	6	0.085161	1	0.082324	1.0845	1	0.46102	0.25
Nitrogen metabolism	6	0.085161	1	0.082324	1.0845	1	0.46102	0.25
Biosynthesis of unsaturated fatty acids	36	0.51097	2	0.090621	1.0428	1	0.47576	0
Ascorbate and aldarate metabolism	8	0.11355	1	0.1083	0.96538	1	0.5062	0.2
Valine, leucine and isoleucine degradation	40	0.56774	2	0.10847	0.96468	1	0.5062	0.03774
Nicotinate and nicotinamide metabolism	15	0.2129	1	0.19379	0.71267	1	0.85675	0.04762
Histidine metabolism	16	0.2271	1	0.20534	0.68752	1	0.86245	0.06667
Citrate cycle (TCA cycle)	20	0.28387	1	0.25	0.60206	1	1	0.03448
Pyruvate metabolism	22	0.31226	1	0.27142	0.56635	1	1	0.03704
Propanoate metabolism	23	0.32645	1	0.28191	0.54989	1	1	0.03846
Galactose metabolism	27	0.38323	1	0.32245	0.49154	1	1	0
Phosphatidylinositol signaling system	28	0.39742	1	0.33224	0.47855	1	1	0.02128

Porphyrin and chlorophyll metabolism	30	0.42581	1	0.35141	0.45419	1	1	0.03846
Inositol phosphate metabolism	30	0.42581	1	0.35141	0.45419	1	1	0.05
Glycine, serine and threonine metabolism	33	0.46839	1	0.37918	0.42115	1	1	0.02564
Arachidonic acid metabolism	36	0.51097	1	0.40582	0.39167	1	1	0.02778
Arginine and proline metabolism	38	0.53935	1	0.42297	0.37369	1	1	0.025
Tryptophan metabolism	41	0.58194	1	0.44781	0.34891	1	1	0.07692
Purine metabolism	65	0.92258	1	0.6129	0.21261	1	1	0
Steroid hormone biosynthesis	85	1.2065	1	0.71336	0.14669	1	1	0

Supplementary Table S5. Metabolic pathway analysis (MetPA) of studies in human CSF of progressive MS (PPMS, SPMS) patients versus healthy controls. 3 pathways (in bold) are significant (i.e. p<0.05). P values which are significant are highlighted in green.

CSF_Progressive MS vs controls; pathways	Total	Expected	Hits	Raw p	"-log10p"	Holm adjust	FDR	Impact
**Tryptophan metabolism**	41	0.55548	6	9.53E-06	5.021	0.0008003	0.0008003	0.20512
**Sphingolipid metabolism**	21	0.28452	2	0.031451	1.5024	1	1	0.21875
**Lysine degradation**	25	0.33871	2	0.043495	1.3616	1	1	0.1
Pyrimidine metabolism	39	0.52839	2	0.095955	1.0179	1	1	0.0678
Valine, leucine and isoleucine biosynthesis	8	0.10839	1	0.10361	0.98461	1	1	0
Ubiquinone and other terpenoid-quinone biosynthesis	9	0.12194	1	0.11582	0.93623	1	1	0
Vitamin B6 metabolism	9	0.12194	1	0.11582	0.93623	1	1	0
Caffeine metabolism	10	0.13548	1	0.12786	0.89325	1	1	0.14286
Arginine biosynthesis	14	0.18968	1	0.17452	0.75817	1	1	0.0625
Nicotinate and nicotinamide metabolism	15	0.20323	1	0.1858	0.73095	1	1	0.04762
Purine metabolism	65	0.88065	2	0.21902	0.65952	1	1	0.02326
Arginine and proline metabolism	38	0.51484	1	0.40826	0.38906	1	1	0
Valine, leucine and isoleucine degradation	40	0.54194	1	0.42459	0.37203	1	1	0.01887
Tyrosine metabolism	42	0.56903	1	0.44049	0.35607	1	1	0.02326
Aminoacyl-tRNA biosynthesis	48	0.65032	1	0.48571	0.31362	1	1	0.03448

Supplementary Table S6. Metabolic pathway analysis (MetPA) of studies in mouse models of CNS demyelinating disease in all biofluids and brain tissue. 6 pathways (in bold) are significant (i.e. p<0.05). P values which are significant are highlighted in green.

Metabolic pathways	Total	Expected	Hits	Raw p	"-log10p"	Holm adjust	FDR	Impact
**Biosynthesis of unsaturated fatty acids**	36	0.47809	6	3.69E-06	5.4326	0.0003102	0.0003102	0
**Valine, leucine and isoleucine biosynthesis**	8	0.10624	3	0.00010768	3.9679	0.0089375	0.0045226	0.25
**Glycerophospholipid metabolism**	36	0.47809	4	0.0010158	2.9932	0.083295	0.028442	0.24999
**Linoleic acid metabolism**	5	0.066401	2	0.0016368	2.786	0.13258	0.034372	1
**Arachidonic acid metabolism**	36	0.47809	3	0.010819	1.9658	0.86548	0.18175	0.41667
**Valine, leucine and isoleucine degradation**	40	0.53121	3	0.01447	1.8395	1	0.20258	0.05661
Glycine, serine and threonine metabolism	34	0.45153	2	0.072945	1.137	1	0.87535	0.02381
Phenylalanine metabolism	12	0.15936	1	0.14873	0.82761	1	1	0
alpha-Linolenic acid metabolism	13	0.17264	1	0.16012	0.79555	1	1	0.125
Glycosylphosphatidylinositol (GPI)-anchor biosynthesis	14	0.18592	1	0.17137	0.76605	1	1	0.125
Glycerolipid metabolism	16	0.21248	1	0.19345	0.71344	1	1	0.09091
Pantothenate and CoA biosynthesis	19	0.25232	1	0.22551	0.64683	1	1	0.05556
Sphingolipid metabolism	21	0.27888	1	0.24621	0.60869	1	1	0.03125
Glycolysis / Gluconeogenesis	26	0.34529	1	0.29569	0.52916	1	1	0.02857
Arginine and proline metabolism	38	0.50465	1	0.40215	0.39561	1	1	0.025
Steroid biosynthesis	42	0.55777	1	0.4341	0.36241	1	1	0.02564
Aminoacyl-tRNA biosynthesis	48	0.63745	1	0.47901	0.31966	1	1	0.03448
